# Therapeutic evaluation of homeopathic treatment for canine oral papillomatosis

**DOI:** 10.14202/vetworld.2020.206-213

**Published:** 2020-01-31

**Authors:** P. Albert Arockia Raj, Selvaraj Pavulraj, M. Asok Kumar, S. Sangeetha, R. Shanmugapriya, S. Sabithabanu

**Affiliations:** 1 Veterinary Dispensary, Department of Animal Husbandry, Radhapuram, Villupuram, Tamil Nadu, India; 2 Institute of Virology, Center for Infectious Medicine, Freie University of Berlin, Robert-von-Ostertag-Street 7-13, Berlin - 14163, Germany; 3 Division of Pathology, Indian Veterinary Research Institute, Bareilly, Uttar Pradesh, India; 4 Madras Veterinary College, Chennai, Tamil Nadu, India

**Keywords:** Graphites, homeopathy, oral papillomatosis, Psorinum, Sulfur, *Thuja*

## Abstract

**Aim:**

A study was conducted to evaluate the ameliorative potential of homeopathic drugs in combination (Sulfur 30C, *Thuja* 30C, Graphites 30C, and Psorinum 30C) in 16 dogs affected with oral papillomatosis which was not undergone any previous treatment.

**Materials and Methods:**

Dogs affected with oral papillomatosis, which have not undergone any initial treatment and fed with a regular diet. Dogs (total=16) were randomly divided into two groups, namely, homeopathic treatment group (n=8) and placebo control group (n=8). Random number table was used for allocation. Homeopathic combination of drugs and placebo drug (distilled water) was administered orally twice daily for 15 days. Clinical evaluation in both groups of dogs was performed by the same investigator throughout the period of study (12 months). Dogs were clinically scored for oral lesions on days 0, 5, 7, 10, 15, 20, 25, 30, 45, 60, 90, 120, and 150 after initiation of treatment.

**Results:**

The homeopathic treatment group showed early recovery with a significant reduction in oral lesions reflected by clinical score (p<0.001) in comparison to placebo-treated group. Oral papillomatous lesions regressed in the homeopathic group between 7 and 15 days, whereas regression of papilloma in the placebo group occurred between 90 and 150 days. The homeopathic treated group was observed for 12 months post-treatment period and no recurrence of oral papilloma was observed.

**Conclusion:**

The current study proves that the combination of homeopathy drugs aids in fastening the regression of canine oral papilloma and proved to be safe and cost-effective.

## Introduction

Papilloma is a benign, exophytic, neoplastic proliferation of squamous epithelium caused by infection with papillomavirus; a non-enveloped double-stranded DNA virus which has a tropism for mucous membrane and skin [1]. Papillomavirus infections were associated with the various neoplastic, hyperplastic, and dysplastic condition in human and animals [2]. Canine oral papillomatosis (COP) is the most common, self-limiting neoplastic disease of dogs caused by canine oral papillomavirus (COPV). Dogs of <4 years old are commonly affected and there is no difference in the prevalence of COP among different breeds of dogs with no gender predisposition [3]. COP is characterized by high morbidity and low mortality with good prognosis, and animal stays immune for the rest of life [4]. Older dogs, puppies, and immunocompromised dogs are more susceptible to contracting the papillomavirus. COPV affects dog’s oral mucous membrane (dogs younger than 2 years of age) and older dog’s skin [5-8]. Various therapeutic approaches are available for treating COP, such as autoimmune therapy, laser therapy, surgical therapy, cryotherapy, photodynamic therapy, intravenous injection of vincristine sulfate/taurolidine/immunoregulin, intramuscular injections of anthiomaline (lithium antimony thiomalate), oral administration of azithromycin, and topical application of fluorouracil/*Thuja* [9].

Recently, homeopathic treatment approaches gaining significant interest among physicians and veterinarians. Homeopathy is a system of treatment where minute quantities of substances are serially diluted and potentiated, which in larger doses might produce symptoms similar to those of the ailment being treated [10]. Preparation of homeopathic medicines includes preparation of mother tinctures and potencies, indicated by the letter “C” (Centesimal). Doses of homeopathic medicines are not expressed in amount as mg and instead they are expressed in potency or C. Potency is the optimum power of medicine or remedy which given in minimum dose at specified intervals, depending on the nature of the disease and the susceptibility of the subject would cure the sickness in minimum time [11]. It is usually used to refer to the degree of dilution that a homeopathic remedy has undergone in its manufacturing process. This is indicated by the number and letter listed after the name of the remedy. For example, sulfur 30C has undergone 30 steps of dilution, each step has been a one to one hundred dilution (indicated by the letter “C” meaning centesimal) [12,13]. Homeopathic drugs are recently used in treating conditions such as canine atopic dermatitis [14], canine babesiosis [15], idiopathic epilepsy in dogs [16], bovine mastitis [17,18], endometritis in dairy cows [19], diarrhea in neonatal piglets [20], and bovine papillomatosis [21] in animals.

With this background, the present study was aimed to evaluate the therapeutic effectiveness of homeopathic medicines, namely, Sulfur 30C, *Thuja* 30C, Graphites 30C, and Psorinum 30C in combination against COP.

## Materials and Methods

### Ethical approval

Necessary approval for the clinical trial was obtained from Tamil Nadu Animal Husbandry Department (Villupuram), (18/2015). Signed consent statement was collected from each dog owner before initiation of the clinical trial.

### Source of dogs and obtaining owner consent for experimentation

The study was performed at Veterinary Dispensary, Radhapuram, Villupuram, India, between January 1, 2016, and January 1, 2017. A total of 259 canine cases were reported during the period, of which 16 dogs were diagnosed as COP based on lesions in the oral cavity. Consent was obtained from respective animal owners before the dogs were subjected to placebo and treatment with homeopathic drugs. The details of the dogs under study are listed in Table-1.

**Table 1:** Details of dogs that were used in the experimental study.

S. No.	Breed	Sex	Age in months	Group
1.	Nondescript	Male	9	Group A
2.	Nondescript	Male	5	
3	Labrador	Female	8	
4.	Nondescript	Female	5	
5.	German shepherd	Male	6	
6.	Nondescript	Male	5	
7.	Doberman	Male	9	
8.	Nondescript	Male	3	
9.	Labrador	Female	9	Group B
10.	Nondescript	Male	5	
11.	German shepherd	Female	4	
12.	Nondescript	Female	7	
13.	Nondescript	Male	6	
14.	Labrador	Male	6	
15.	Nondescript	Female	7	
16.	Pomeranian	Female	8	

Group A=Control group treated with placebo, Group B=Homeopathic group treated with a combination of Sulfur 30+Graphites 30+Thuja 30+Psorinum 30

### Clinical examination

The location, size, and shape of the papillomatosis lesions were recorded and photographed.

### Drugs

Four homeopathy drugs, namely, Graphites, Psorinum, Sulfur, and *Thuja* with 30C potency (Schwabe International GmbH, Germany) were procured from a local dealer in India (Mashan Distributors, Chennai).

In veterinary practice, homeopathic preparations of Sulfur, Graphites, Psorinum, and *Thuja* were used in the form of 30C potency for treating dermatological cases [22]. Thus, all the four homeopathy drugs were used in the strength of 30C potency and given orally at 2 drops\5 kg body weight (BW) (Graphites 30+Sulfur 30+Psorinum 30+*Thuja* 30) each. Distilled water was used as a placebo drug for the corresponding group.

### Study design

A prospective, parallel, randomized, double- blinded, and placebo-controlled study design was planned for this experiment by the investigator as described previously [23]. Briefly, all 16 dogs were randomly divided into two groups with eight dogs in Group A (placebo control group) and eight dogs each in Group B (homeopathic drugs treatment group). Random allocation was done using tables of random numbers. The person who allocated the dogs into two groups was blinded from the knowledge of treatment to avoid allocation bias in the assignment of treatments. The person involved in sequence generation by randomization was not allowed for the allocation of dogs into treatment groups. The allocations of dogs into treatment and placebo groups were kept blind to the knowledge of both animal owners and investigators. The placebo and combination of homeopathic drugs were filled in similar bottles and consecutively numbered for each dog according to the randomization schedule. All the dogs in Group A were administered with distilled water orally at 8 drops\5 kg BW. All the Group B dogs were treated with homeopathic drugs in combination (Sulfur 30, *Thuja* 30, Psorinum 30, and Graphites 30) orally. Each homeopathy drug was given at the rate of 2 drops\5 kg BW. Homeopathic drugs in combination and placebo were given twice daily for 15 days in 12 h interval. The final dose with homeopathic drug combination used for this current experiment was arrived based on the pilot study (data not shown). Vaccinations or any other supplementary treatment were not permitted during the study period. All the dogs were fed with a regular diet. The main inclusion criterion for the selection of dogs for the experiment was the dogs which have not undergone any initial treatment to find the potential effectiveness of the homeopathic drug in treating COP.

### Clinical scoring

The clinical scoring of papilloma was performed as described previously [23]. Evaluation of the clinical signs and progression of treatment were compared in both groups of dogs. Dogs were clinically scored by examining the oral lesions on 0, 5, 7, 10, 15, 20, 25, 30, 45, 60, 90, 120, and 150 days post-treatment. The investigator is blinded to the type of treatment given. The dogs were clinically scored for severity and regression of papilloma lesions which ranged from scores 0 to 3 (absent-0, mild-1, moderate-2, and severe-3), +1 for <10 number of papillomas, and +2 >10 number of papillomas. Dogs in both groups were examined for the presence of any adverse effect of the treatment. All the dogs were observed for 12 months for any recurrence of papilloma.

### Sample collection for histological and molecular analysis

Physical examination, complete blood count, and serum biochemistry were performed for all the dogs at different time points. Biopsy samples were taken from papillomatous lesions on 0^th^ and 7^th^ days post-treatment from dogs under sedation (Atropine at 0.04 mg/kg BW, Xylazine at 1 mg/kg BW). Biopsy specimens were collected from the buccal region and were samples fixed in 10% neutral buffered formalin, and tissues were processed by routine paraffin-embedding technique. Briefly, 4-5 µm thick sections were cut and stained by routine hematoxylin and eosin for detailed histopathological studies. In addition, piece of biopsy tissues collected from each animal was stored at −70°C for nucleic acid-based molecular studies.

### DNA isolation and polymerase chain reaction (PCR)

Biopsy tissue samples for viral DNA extraction were collected on the 0^th^ day for PCR confirmation of canine papillomavirus infection. Collected papillomatous tissue samples were homogenized with the help of tissue homogenizer and viral DNA extracted using QIA amp mini kit (Qiagen, Germany) as per manufacturer’s instructions. Isolated DNA samples were stored at −70°C until further use. Primers were designed based on the reported sequence of canine papillomavirus 1 (Accession number: L22695.1) targeting L1 open reading frame. PCR was performed on isolated DNA samples using a set of primer sequence, forward primer: 5’-ctgaaaaagaaagacttgtttg-3’ and reverse primer: 5’-attgccccatgcaatcccatta-3’. Viral DNA samples were amplified using PCR in a final volume of 25 µl containing 5 µl 5× Phusion^®^ HF buffer, 0.5 µl of 10 mM dNTPs, 1.25 µl of 10 mM forward primer, 1.25 µl of 10 mM reverse primer, 0.25 µl Phusion^®^ DNA polymerase, 5 µl of isolated DNA sample, and 11.75 µl of nuclease-free water. Thermo cycling was performed in Eppendorf^®^ Thermo Cycler (6331 Nexus gradient Mastercycler Thermal Cycler, Germany) with the following cycling condition: One cycle at 98°C for 30 s, then 35 cycles of 10 s at 98°C, 30 s at 55°C, and 30 s at 72°C and final extension at 72°C for 10 min. Five microliters of PCR product obtained after amplification was run on 1.5% agarose gel in 0.5× Tris-acetate-EDTA buffer containing 1 μl of 1% ethidium bromide at 100 volts for 1 h along with 1 kb plus marker (Thermo Fisher Scientific^™^). Results were documented using UVP-GDS-2000^®^ gel documentation system. Visualization of a band of 707 base pair (bp) size amplified product was the confirmation for the presence of canine papillomavirus nucleic acid in the tissue sample.

### Statistical analysis

Mann–Whitney U-test was used to analyze the difference in the clinical score observed between the two groups. Wilcoxon test was used to compare the clinical success rate of each treatment group with the examination days. The level of significance was defined as p<0.05.

## Results

### Clinical signs

Clinical examination of dogs revealed almost normal physiological activity (body temperature, heart rate, respiration rate, and conjunctival mucous membrane) with the exception of a few animals showed mild-to-moderate anorexia because of oral papillomatous lesions. Results of the complete blood count and serum chemistry of all dogs were within the normal range (data not shown).

### Clinical examination

**Figure-1:**
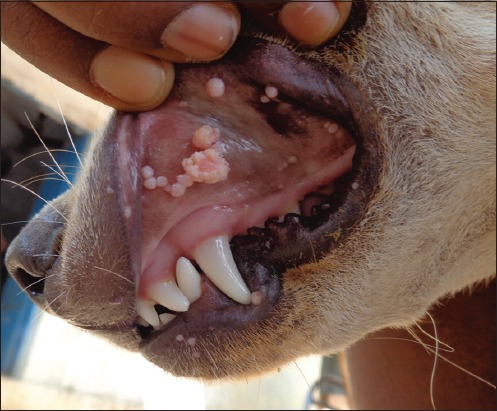
In Group B, variable-sized proliferative papillomas in the oral cavity, lips and mucocutaneous junction on day 0 of treatment.

Examination of the oral cavity revealed numerous papillomatous growths protruding from the surface of oral mucous membranes of infected dogs. Papilloma noticed as small smooth papules (as few millimeters in diameter) to cauliflower-like growth/warts of varied sizes (up to 2-3 cm in diameter). Warts were grayish-white, pink, and black in color due to pigmentation. Grossly, lesions were characterized by hard verrucose, proliferative, and hyperkeratotic usually variable sized, multiple, sessile, and also single and cluster (Figure-1). The papilloma had smooth and rough, jagged, and crumbling surfaces which can be removed but causes bleeding. Papillomatous lesions had narrow base with a wide projection on the mucosal surface. Cut sections revealed multiple fingers like projections. A tentative diagnosis of COP was made based on clinical examination. It should be noted that there was no associated dermatological abnormality in all the dogs except oral papilloma lesions.

In Group A, none of the dogs treated with placebo showed regression of oral lesion on 7 days of post-treatment. In Group B, treatment with homeopathic drug combination resulted in complete regression of oral lesion in 7 days (4 of 8 dogs). On the 10^th^ day of post-treatment, none of the dogs in Group A showed regression but in Group B, three more dogs showed complete regression. On the 15^th^ day of post-treatment, still, none of the dogs in Group A showed regression of oral lesion but in Group B, the remaining one dog showed complete regression. On contrary to the treatment group, the placebo treatment group showed regression from the 90^th^ day onward. Shortly, three dogs showed complete regression of papilloma around 90^th^ day and two dogs on 120^th^ day after post-placebo treatment. Remaining three dogs in the placebo group showed regression around 150^th^ day.

### Histopathological examination

**Figure-2:**
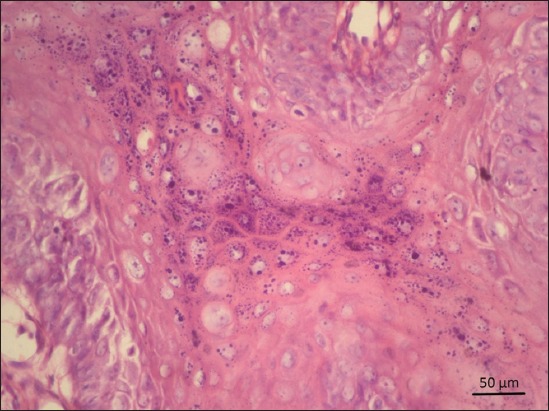
In Group B, at day 0 of treatment, hyperplasia of stratum corneum with severe infiltrations of keratohyaline granules in the cytoplasm of proliferating epithelial cells. Hematoxylin and eosin 400×.

**Figure-3:**
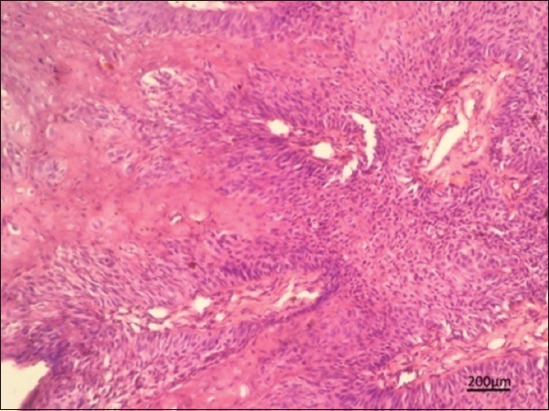
In Group B, hyperplasia of stratum corneum of stratified squamous epithelium with the central fibrous core on day 0 of treatment. Hematoxylin and eosin 100×.

**Figure-4:**
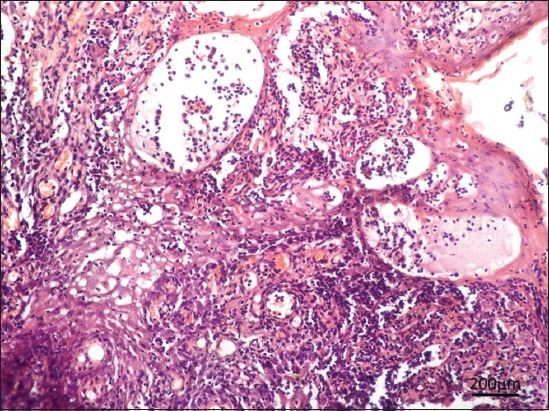
In group B, 5^th^ day of homeopathic treatment showed reduced hyperplasia, neovascularization, parakeratosis with severe infiltration of mononuclear cells in connective tissue stroma. Hematoxylin and eosin 100×.

Histopathological examination of the papillomatous lesion before treatment was characterized by severe hyperplasia of stratum corneum layer of stratified squamous epithelium, multiple rete pegs formation with central fibrous core (Figures-2 and 3). Moderate to severe infiltrations of keratohyalin granules were observed in the cytoplasm of proliferating epithelial cells. The presence of intranuclear inclusions in the keratinocytes was noticed in a few sections. Other associated findings noticed were parakeratosis and eosinophilic cytoplasmic inclusions (koilocytes). Tissue sections of dogs from 5^th^ day of homeopathic treatment revealed reduction in hyperplastic changes, development of uniform cells without anaplastic changes, neovascularization in between fibrous stroma of core, moderate to severe diffuse infiltration of the mononuclear cells in the connective tissue stroma, loss of keratin layer as a whole, and a focal area of keratinization with nucleus in stratum corneum were noticed suggesting regeneration (Figure-4).

### PCR

**Figure-5:**
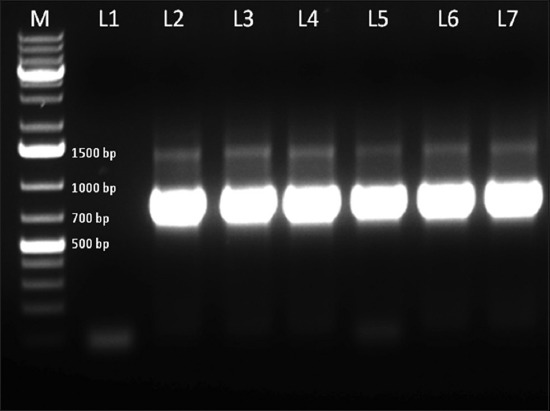
Polymerase chain reaction (PCR) amplification of canine papillomavirus DNA isolated from papillomatous tissue from dogs. PCR product of 707 base pair product confirms positive amplification. M – 1 kb plus marker, L1 - L7: PCR product for tissue samples. L1: Nontemplate negative control; L2-L7: Oral papilloma samples.

PCR analysis revealed the association of canine papillomavirus in all dogs with papillomatous oral lesions. PCR amplification of canine papillomavirus DNA isolated from papillomatous tissue from dogs showed an amplicon size of 707 bp (Figure-5).

### Clinical score

**Figure-6:**
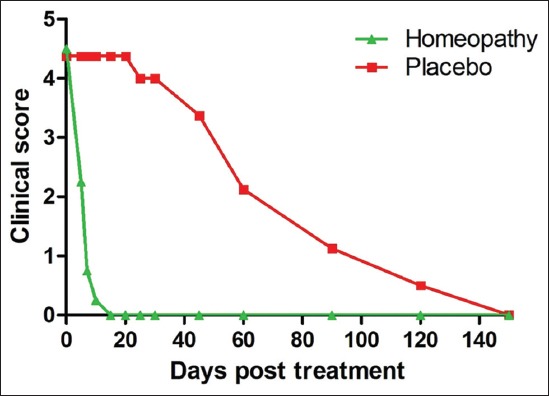
Comparison of clinical scores between Group A (control) and Group B (homeopathic group) during 150 days of the experiment.

**Figure-7:**
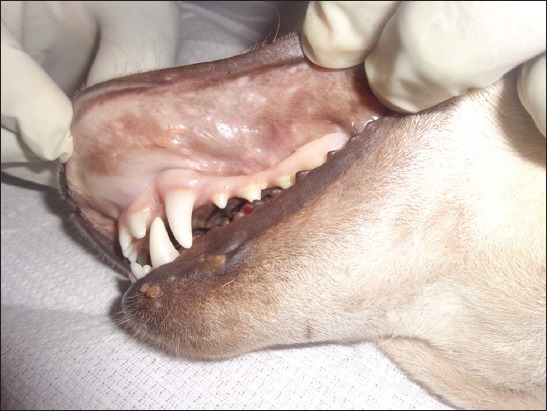
Regression of papilloma lesions on the 5^th^ of homeopathic drug treatment.

On day 0, the clinical scores of both groups were the same. The mean clinical score of dogs belonging to Group A and Group B is presented in Table-2 and Figure-6. In Group A, lesion score for papilloma remained the same (4.375) on 5, 7, 10, and 15 days post-treatment (maximum score is 5). In contrast, lesion score in Group B was only 2.25, 0.75, and 0.25 on 5, 7, and 10 days post-treatment. Further, no lesion observed on day 15 in Group B. Homeopathic treatment (Group B) showed early regression of papilloma lesion on day 5 (Figure-7) with significantly lower clinical score than placebo group (p<0.05) and on days 10, 15, 20, 25, 30, 45, and 60, (p<0.001). In contrast, Group A showed regression of papilloma with a reduction of clinical score from the 90^th^ day (p<0.05) in score compared to baseline and complete regression by the 150^th^ day. The homeopathic drug combination-treated Group (B) showed significant improvement throughout the observation period from days 5 (p<0.05) to 150 (p<0.001) in scores compared to baseline.

**Table-2:** Clinical score received from the dogs affected with oral papilloma in Group A (control group) and Group B (homeopathic treatment group) during 150 days of observation (mean±standard deviation).

Days	Group A (Mean±Standard deviation)	Group B (Mean±Standard deviation)	p-value^a^
0	4.375±0.517	4.5±0.534	0.5
5	4.375±0.517	2.25±1.164	0.007
7	4.375±0.517	0.75±1.035	<0.001
10	4.375±0.517	0.25±0.707	<0.001
15	4.375±0.517	0	<0.001
20	4.375±0.517	0	<0.001
25	4±0.925	0	<0.001
30	4±0.925	0	<0.001
45	3.375±0.916	0	<0.001
60	2.125±1.125	0	<0.001
90	1.125±1.125	0	0.013
120	0.5±0.755	0	0.100
150	0	0	1.000

a Significance of difference between control and treatment group. p<0.05 is considered as statistically significant

## Discussion

Despite autoregression, treating oral papilloma cases are very crucial because of the physical difficulty the animal faces while taking food, restriction from social interaction with other dogs, anorexia, drooling, halitosis, bleeding, secondary bacterial infections, and purulent discharge near papillomas [4]. Various therapeutic regimes were used to alleviate COP. External application of apple cider vinegar, pod wart, and petroleum jelly [9] *Thuja* *occidentalis* [24], imiquimod, and 5-fluorouracil [8]; oral administration of levamisole [3], cimetidine [25], interferon alfa [7], human recombinant interferon-α 2a [26], *T. occidentalis* [27], acyclovir [28], etretinate [7], and azithromycin [23]; subcutaneous injection of tarantula cubenis extract [29], feline recombinant interferon-ω [30], autogenous vaccine [31], and *T. occidentalis* [32]; intramuscular injection of lithium antimony thiomalate [9]; intramuscular injection of *Propionibacterium* acnes [33]; intralesional administration of interferon alfa [7]; and intravenous injection of vincristine sulfate and immunoregulin [31], and taurolidine [3] were reported. Injectable interferon directly stimulates the immune system thereby helps to resolve warts. Non or semi-invasive approaches such as surgical excision, debulking of exophytic lesion with electro or radiosurgery or by sharp resection, electrocautery, cryotherapy, crushing of warts to stimulate immunity [9], crushing laser or cryosurgery [8], photodynamic therapy [34], and spontaneous regression and autoimmune therapy [35] were also reported. COP can be treated with combination of inactivated autogenous vaccine, chemotherapy, and application of *T. occidentalis* in a dog [36]. A recombinant COPV vaccine shows promising results in COP cases which were not responded to treatment [1].

Treatment such as surgical removal and vaccination may increase the size of residual warts and prolong the course of the disease. Surgical removal becomes difficult when the extend of papilloma lesion is more [32]. The relationship between clinical resolution and surgical intervention is difficult to determine due to the frequent spontaneous regression of this papilloma. Treatment is not guaranteed through surgical removal of warts as the papillomavirus is still in the dog’s body leading to latent infection and increased recurrence [37]. Anesthesia is required for surgical removal of warts which make the surgical way of treating papilloma riskier in addition to special care and high cost. Laser and cryotherapy are used in the ablation of papilloma but multiple treatments are generally needed [38]. Autogenous vaccine and immune modulation drugs such as levamisole and thiabendazole are used but proof for their efficiency is lacking [7]. Systemic or local chemotherapy drugs such as vincristine, vinblastine, cyclophosphamide, methotrexate, chlorambucil, and doxorobucin lead to inefficient or controversial results in the majority of papilloma cases, with longer duration of the treatment period and varying degrees of success [3,34].

Homeopathy is an alternative therapy available at affordable prices and becoming increasingly popular in human medicine in developing countries that have negligible adverse effect with the use [39]. Agnihotri *et al*. [9] reported that treating canine oral papilloma with *Thuja* alone for 2 months was not effective. Combinations of Sulfur 30+*Thuja* 30+Graphites 30+Psorinum 30 have been selected for the treatment in our experiment. These homeopathy drugs have been used in human medicine for long. None of the homeopathic drugs used in this experiment have been proven to cause any side effect. It has been already proved that *Thuja* (*T. occidentalis*) has immunomodulatory and antiviral properties which cause B and T lymphocyte proliferation and differentiation into CD4+ cells and induces production of interleukin (IL)-2, IL-1, IL-6, tumor necrosis factor-α, and interferon-γ production *in vitro* and *in vivo* [40]. Sulfur 30 promotes healthy skin condition which heals damaged skin, Graphites 30 is a powerful anti-psoric and commonly used against eruptions in the skin and mucosal surfaces in humans and Psorinum 30 also has anti-psoric activity [41]. Sulfur was used to treat pruritus associated with atopic dermatitis in dogs [14]. Graphites were used to treat hypertrophic burn scars successfully in humans [42] and canine demodicosis [43]. Combination of Sulfur, Psorinum, and Graphites with other homeopathic drugs was used in dogs for treating various skin conditions such as seborrhea, pruritus, allergic dermatitis, and Hot Dog Syndrome [44]. Sulfur, Psorinum, and Graphite in combination are used to treat canine mange [45]. Madrewar and Glencross [22] have stated the use of different combinations of homeopathy drugs against all kinds of dermatitis, ringworm, wet or dry eczema and to remove warts of all types on any part of the animal body. The combination of homeopathic drugs (Sulfur 30+*Thuja* 30+Graphites 30+Psorinum 30) was found to be very effective in the treatment of COP; as a result, complete regression of papilloma lesions observed between 7 and 15 days after initiation of treatment. Further, no adverse effect was observed during the course and after the treatment. No recurrence of papilloma was observed during the follow-up period of 12 months in all treated dogs. It proves that these combinations Sulfur 30+*Thuja* 30+Graphites 30+Psorinum 30 could be used as a potential non-invasive method for the successful treatment of COP which would be alternate to surgical removal or using toxic drugs like vincristine.

## Conclusion

The result of this investigation proves that the combination of homeopathic drugs (Sulfur 30+*Thuja* 30+Graphites 30+Psorinum 30) offers an attractive, non-invasive and most economical way of treating COP. A combination of homeopathic drugs is a novel approach for treating canine oral papilloma and further studies are needed to elucidate the use of homeopathic combination as a veterinary oncological therapeutics and to explore the mechanism of action of these homeopathic drugs in ameliorating oral papilloma.

## Authors’ Contributions

PAAR designed the experiment, sorted dogs into two groups and wrote most parts of the manuscript. SP wrote the discussion and performed analysis of the data; MAK performed histopathology and PCR. SS and RS performed clinical evaluation and analysis. SSab performed randomization, blinding, and literature search. All authors read and approved the final version of the manuscript.
